# 2-(5-Iodo-2-oxoindolin-3-yl­idene)hydrazinecarbo­thio­amide including an unknown solvate

**DOI:** 10.1107/S1600536814010782

**Published:** 2014-05-17

**Authors:** Viviane Conceição Duarte de Bittencourt, Vanessa Carratu Gervini, Juliano Rosa de Menezes Vicenti, Jecika Maciel Velasques, Priscilla Jussiane Zambiazi

**Affiliations:** a Escola de Química e Alimentos, Universidade Federal do Rio Grande, Av. Itália, km 08, Campus Carreiros, 96203-900, Rio Grande, RS, Brazil; b Departamento de Química, Universidade Federal de Santa Maria, Av. Roraima, Campus, 97105-900, Santa Maria, RS, Brazil

## Abstract

The mol­ecule of the title compound, C_9_H_7_IN_4_OS, is almost planar (r.m.s. deviation = 0.0373 Å). In the mol­ecule, N—H⋯N and N—H⋯O hydrogen bonds generate, respectively, *S*(5) and *S*(6) ring motifs. In the crystal, mol­ecules are linked *via* N—H⋯O hydrogen bonds, forming chains propagating along [010]. These chains are linked *via* S⋯I contacts [3.4915 (16) Å], forming sheets lying parallel to (100). A region of disordered electron density, probably a disordered tetra­hydro­furan solvent mol­ecule, was treated using the SQUEEZE routine in *PLATON* [Spek (2009). *Acta Cryst.* D**65**, 148–155]. The formula mass and unit-cell characteristics were not taken into account during refinement.

## Related literature   

For the synthesis, see: Chiyanzu *et al.* (2003 ▶). For applications, see: Silva *et al.* (2001 ▶); Chiyanzu *et al.* (2003 ▶). For similar structures, see: de Bittencourt *et al.* (2014 ▶); Bandeira *et al.* (2013 ▶); de Oliveira *et al.* (2012 ▶). For S⋯I inter­actions, see: Auffinger *et al.* (2004 ▶).
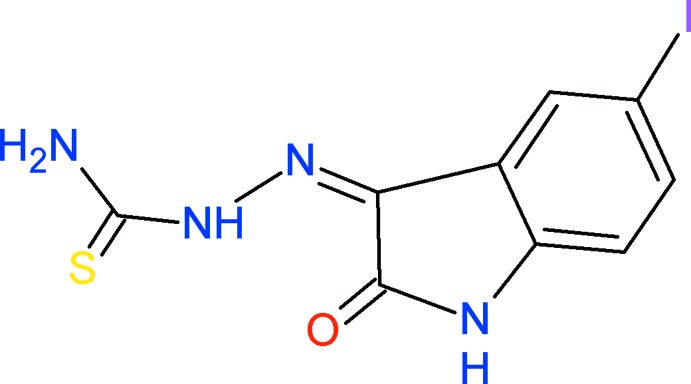


## Experimental   

### 

#### Crystal data  

C_9_H_7_IN_4_OS
*M* *_r_* = 346.15
Monoclinic, 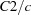

*a* = 33.765 (5) Å
*b* = 4.4569 (5) Å
*c* = 19.977 (3) Å
β = 123.100 (4)°
*V* = 2518.4 (6) Å^3^
*Z* = 8
Mo *K*α radiation
μ = 2.69 mm^−1^
*T* = 100 K
1.15 × 0.10 × 0.09 mm

#### Data collection  

Bruker APEXII CCD diffractometer
Absorption correction: numerical (*SADABS*; Bruker, 2009 ▶) *T*_min_ = 0.701, *T*_max_ = 0.777
18679 measured reflections
2892 independent reflections
2577 reflections with *I* > 2σ(*I*)
*R*_int_ = 0.036

#### Refinement  

*R*[*F* ^2^ > 2σ(*F* ^2^)] = 0.028
*wR*(*F* ^2^) = 0.062
*S* = 1.05
2892 reflections
161 parameters
H atoms treated by a mixture of independent and constrained refinement
Δρ_max_ = 1.50 e Å^−3^
Δρ_min_ = −1.52 e Å^−3^

### 

Data collection: *APEX2* (Bruker, 2009 ▶); cell refinement: *SAINT* (Bruker, 2009 ▶); data reduction: *SAINT*; program(s) used to solve structure: *SHELXS97* (Sheldrick, 2008 ▶); program(s) used to refine structure: *SHELXL97* (Sheldrick, 2008 ▶); molecular graphics: *DIAMOND* (Brandenburg, 2006 ▶); software used to prepare material for publication: *publCIF* (Westrip, 2010 ▶) and *PLATON* (Spek, 2009 ▶).

## Supplementary Material

Crystal structure: contains datablock(s) I, New_Global_Publ_Block. DOI: 10.1107/S1600536814010782/bg2528sup1.cif

Structure factors: contains datablock(s) I. DOI: 10.1107/S1600536814010782/bg2528Isup2.hkl

Supporting information file. DOI: 10.1107/S1600536814010782/bg2528Isup3.cml

CCDC reference: 1002200

Additional supporting information:  crystallographic information; 3D view; checkCIF report

## Figures and Tables

**Table 1:** Hydrogen-bond geometry (Å, °)

*D*—H⋯*A*	*D*—H	H⋯*A*	*D*⋯*A*	*D*—H⋯*A*
N1—H12⋯N3	0.85 (4)	2.28 (4)	2.633 (3)	105 (3)
N2—H21⋯O1	0.83 (4)	2.07 (4)	2.725 (3)	135 (3)
N4—H41⋯O1^i^	0.77 (3)	2.04 (4)	2.809 (3)	178 (3)
N1—H11⋯S1^ii^	0.79 (3)	2.66 (4)	3.448 (3)	170 (3)

Symmetry codes: (i) 
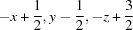
; (ii) 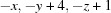.

**Fig. 1.**
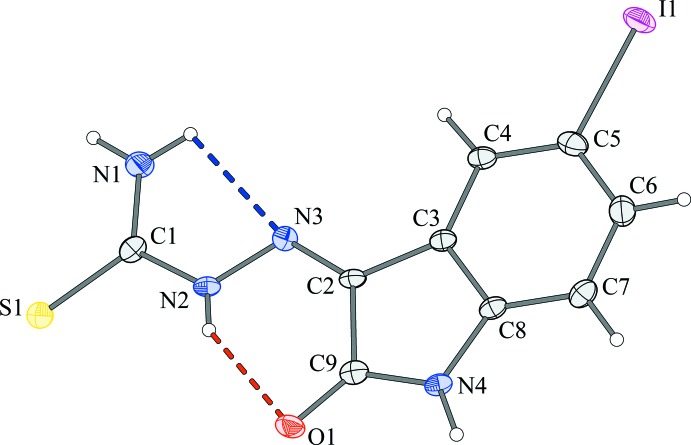
Asymmetric unit of the title compound showing intramolecular hydrogen bond interactions represented with dashed lines. Ellipsoid probability: 50%.

**Fig. 2.**
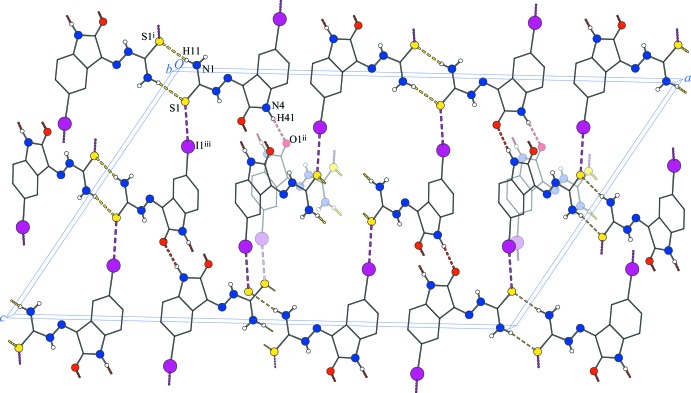
Packing diagram of the title compound showing hydrogen bond network generated through interactions represented as dashed lines. Some hydrogen atoms were omitted for clarity. Symmetry codes: (i) –x, 2–y, –z; (ii) 1/2–x, -1/2 + y, 1/2–z; (iii) x, –y, 1/2 + z.

**Fig. 3.**
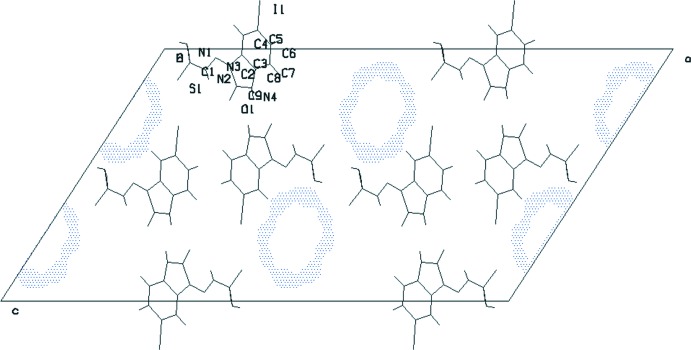
Plot generated by PLATON showing solvent accessible voids positions in the unit cell as dashed delimited circles.

## supplementary crystallographic information

### 1. Comment

Isatins and their derivatives are described as compounds with technological applications, such as organic analytical chemistry and pigments and dyes (Silva *et al.*, 2001). Particularly, halogen substituted isatins containing thiosemicarbazone fragment demonstrated biological activity against some trypanosomes (cruzain and rhodesain) and malaria (falcipain-2) parasites (Chiyanzu *et al.*, 2003). In this paper, we describe the crystal structure of 2-(5-Iodo-2-oxoindolin-3-ylidene)-hydrazinecarbothioamide, C_9_H_7_ON_4_SI. There is one molecule in the asymmetric unit (Figure 1), and it is very similar to that of the Cl analogue previously published by us (de Bittencourt *et al.*, 2014). The molecule is almost planar (Bandeira *et al.*, 2013; de Oliveira *et al.*, 2012), displaying a r.m.s. deviation of 0.0373 Å for all fitted non-hydrogenoid atoms (with maximum deviation of 0.1057 (12) observed for S1 atom). Although the same intramolecular S(5) and S(6) ring motifs are verified (Table 1), the crystal packing differs totally when compared with the chloro substituted analog. In the present case, co-operative dimmers are formed through N1—H11···S1^i^ interactions displaying distances of 2.67 (4) Å. The crystal structure is expanded along the [010] crystallographic direction through N4—H41···O1^ii^ interactions with distances of 2.08 (4) Å, generating a *one-dimensional polymer-like* motif. One of the most interesting features are the S···I^iii^ contacts which further connects the molecules along the [001] crystallographic direction, presenting distances of 3.4915 (16) Å (Figure 2, symmetry codes: (i) –*x*, 2–*y*, –*z*; (ii) 1/2–*x*, -1/2 + *y*, 1/2–*z*; (iii) *x*, –*y*, 1/2 + *z*). Such S···I short interactions are related to be present in sulfur-containing proteins like cysteine and methionine (Auffinger *et al.*, 2004), being mentioned to play an important hole in biological systems. As a second interesting feature, it was noted disordered solvent molecules occupying accessible voids of 83 Å^3^, suggestive of tetrahydrofuran, which was indeed used for crystallization. Due to this, collected data was treated using the *SQUEEZE* routine of the *PLATON* software (Spek, 2009) in order to remove solvent electronic density. The new *HKP* file generated was then used for further refinement of the final solvent free crystal structure. For solvent-voids position assignement in the unit cell, a solvent plot calculated by *PLATON* is shown in Figure 3.

### 2. Experimental

To 20 ml of ethanol it was mixed 500 mg (1.83 mmol s) of 5-iodo-isatine with 170 mg (1.83 mmol s) of thiosemicarbazide. Then, it was added ten drops of glacial acetic acid and the system was kept under reflux for four hours. After this time, the reaction mixture was cooled and an orange precipitated was filtered off under vacuum and dried at room temperature. m.p. 249–253 °C. Yield: 98%. Crystallization: needle single crystals of the title compound suitable for X-ray diffraction were obtained by slow evaporating a solution containing 30 mg of the product dissolved in 5 ml of tetrahydrofuran.

### 3. Refinement

All H atoms attached to C atoms were positioned with idealized geometry and were refined isotropic with *U*_eq_(H) set to 1.2 times of the *U*_eq_(C). It was used a riding model with aromatic C—H = 0.93 Å. Reflection 200 was omitted due to the large difference observed between *F_o_*^2^ and Fc^2^.

The needle-like single crystals were very weakly diffracting for what an unusually large crystal (1.152mm, with a 0.6 mm collimator) was used.

### Crystal data

**Table:**

C_9_H_7_IN_4_OS	*F*(000) = 1328
*M**_r_* = 346.15	*D*_x_ = 1.826 Mg m^−^^3^
Monoclinic, *C*2/*c*	Melting point: 522 K
Hall symbol: -C 2yc	Mo *K*α radiation, λ = 0.71073 Å
*a* = 33.765 (5) Å	Cell parameters from 9997 reflections
*b* = 4.4569 (5) Å	θ = 2.4–28.3°
*c* = 19.977 (3) Å	µ = 2.69 mm^−^^1^
β = 123.100 (4)°	*T* = 100 K
*V* = 2518.4 (6) Å^3^	Needle, yellow
*Z* = 8	1.15 × 0.10 × 0.09 mm

### Data collection

**Table:**

Bruker APEXII CCD diffractometer	2892 independent reflections
Radiation source: fine-focus sealed tube	2577 reflections with *I* > 2σ(*I*)
Graphite monochromator	*R*_int_ = 0.036
*φ* and *ω* scans	θ_max_ = 28.3°, θ_min_ = 2.0°
Absorption correction: numerical (*SADABS*; Bruker, 2009)	*h* = −44→44
*T*_min_ = 0.701, *T*_max_ = 0.777	*k* = −3→5
18679 measured reflections	*l* = −26→26

### Refinement

**Table:**

Refinement on *F*^2^	Primary atom site location: structure-invariant direct methods
Least-squares matrix: full	Secondary atom site location: difference Fourier map
*R*[*F*^2^ > 2σ(*F*^2^)] = 0.028	Hydrogen site location: inferred from neighbouring sites
*wR*(*F*^2^) = 0.062	H atoms treated by a mixture of independent and constrained refinement
*S* = 1.05	*w* = 1/[σ^2^(*F*_o_^2^) + (0.0208*P*)^2^ + 10.8261*P*] where *P* = (*F*_o_^2^ + 2*F*_c_^2^)/3
2892 reflections	(Δ/σ)_max_ = 0.001
161 parameters	Δρ_max_ = 1.50 e Å^−^^3^
0 restraints	Δρ_min_ = −1.52 e Å^−^^3^

### Special details

**Table:**

Geometry. All e.s.d.'s (except the e.s.d. in the dihedral angle between two l.s. planes) are estimated using the full covariance matrix. The cell e.s.d.'s are taken into account individually in the estimation of e.s.d.'s in distances, angles and torsion angles; correlations between e.s.d.'s in cell parameters are only used when they are defined by crystal symmetry. An approximate (isotropic) treatment of cell e.s.d.'s is used for estimating e.s.d.'s involving l.s. planes.
Refinement. Refinement of *F*^2^ against ALL reflections. The weighted *R*-factor *wR* and goodness of fit *S* are based on *F*^2^, conventional *R*-factors *R* are based on *F*, with *F* set to zero for negative *F*^2^. The threshold expression of *F*^2^ > σ(*F*^2^) is used only for calculating *R*-factors(gt) *etc*. and is not relevant to the choice of reflections for refinement. *R*-factors based on *F*^2^ are statistically about twice as large as those based on *F*, and *R*- factors based on ALL data will be even larger.

### Fractional atomic coordinates and isotropic or equivalent isotropic displacement parameters (Å^2^)

**Table:**

	*x*	*y*	*z*	*U*_iso_*/*U*_eq_	
C6	0.20209 (11)	0.5198 (5)	0.48073 (18)	0.0190 (6)	
H6	0.2160	0.3772	0.4658	0.023*	
C7	0.22592 (10)	0.6149 (6)	0.55978 (17)	0.0168 (5)	
H7	0.2555	0.5382	0.5981	0.020*	
C5	0.15782 (10)	0.6347 (6)	0.42365 (16)	0.0170 (6)	
H41	0.2442 (12)	0.924 (7)	0.693 (2)	0.016 (8)*	
H21	0.1270 (13)	1.502 (7)	0.631 (2)	0.023 (9)*	
H11	0.0129 (12)	1.770 (7)	0.4600 (19)	0.019 (8)*	
H12	0.0403 (15)	1.560 (9)	0.449 (3)	0.043 (12)*	
I1	0.125098 (8)	0.47387 (4)	0.306159 (11)	0.02521 (7)	
S1	0.06378 (3)	1.88628 (14)	0.62093 (4)	0.01889 (15)	
O1	0.19376 (7)	1.3146 (4)	0.70372 (11)	0.0168 (4)	
N4	0.22091 (9)	0.9605 (5)	0.65344 (15)	0.0145 (5)	
N3	0.11031 (8)	1.3194 (4)	0.53273 (13)	0.0130 (4)	
C9	0.18938 (9)	1.1590 (5)	0.64912 (16)	0.0137 (5)	
C1	0.06744 (9)	1.6773 (5)	0.55453 (16)	0.0140 (5)	
N1	0.03597 (10)	1.6699 (6)	0.47747 (15)	0.0209 (5)	
C3	0.15944 (9)	0.9428 (5)	0.52186 (16)	0.0127 (5)	
N2	0.10679 (9)	1.5041 (4)	0.58282 (15)	0.0145 (5)	
C8	0.20411 (9)	0.8269 (5)	0.57920 (15)	0.0133 (5)	
C2	0.14815 (9)	1.1580 (5)	0.56399 (15)	0.0127 (5)	
C4	0.13579 (10)	0.8471 (5)	0.44356 (16)	0.0143 (5)	
H4	0.1061	0.9222	0.4053	0.017*	

### Atomic displacement parameters (Å^2^)

**Table:**

	*U*^11^	*U*^22^	*U*^33^	*U*^12^	*U*^13^	*U*^23^
C6	0.0251 (17)	0.0160 (11)	0.0226 (17)	0.0021 (10)	0.0173 (14)	0.0005 (9)
C7	0.0115 (15)	0.0176 (11)	0.0198 (15)	0.0015 (9)	0.0076 (12)	0.0034 (9)
C5	0.0212 (16)	0.0181 (11)	0.0129 (15)	−0.0041 (10)	0.0101 (13)	−0.0011 (9)
I1	0.04097 (15)	0.02153 (9)	0.01480 (12)	−0.00098 (7)	0.01630 (11)	−0.00167 (6)
S1	0.0206 (4)	0.0199 (3)	0.0160 (4)	0.0050 (2)	0.0099 (3)	0.0011 (2)
O1	0.0125 (10)	0.0216 (9)	0.0106 (10)	−0.0011 (7)	0.0026 (8)	−0.0027 (7)
N4	0.0091 (13)	0.0177 (10)	0.0095 (13)	0.0006 (8)	0.0005 (11)	0.0010 (8)
N3	0.0119 (12)	0.0143 (9)	0.0125 (12)	−0.0011 (8)	0.0065 (10)	−0.0002 (7)
C9	0.0106 (14)	0.0153 (11)	0.0124 (14)	−0.0029 (9)	0.0044 (12)	0.0013 (9)
C1	0.0101 (14)	0.0154 (11)	0.0160 (15)	−0.0001 (9)	0.0068 (12)	0.0019 (9)
N1	0.0137 (14)	0.0276 (12)	0.0147 (14)	0.0078 (10)	0.0034 (11)	−0.0008 (9)
C3	0.0110 (14)	0.0139 (10)	0.0123 (14)	−0.0017 (9)	0.0057 (12)	0.0019 (8)
N2	0.0130 (13)	0.0168 (10)	0.0091 (13)	0.0007 (8)	0.0030 (10)	−0.0001 (8)
C8	0.0117 (14)	0.0144 (10)	0.0111 (14)	−0.0011 (9)	0.0044 (11)	0.0020 (9)
C2	0.0131 (14)	0.0135 (10)	0.0090 (13)	−0.0025 (9)	0.0043 (11)	0.0008 (8)
C4	0.0119 (14)	0.0159 (11)	0.0110 (14)	−0.0025 (9)	0.0036 (11)	0.0010 (9)

### Geometric parameters (Å, º)

**Table:**

C6—C7	1.390 (4)	N3—C2	1.292 (3)
C6—C5	1.391 (4)	N3—N2	1.350 (3)
C6—H6	0.9300	C9—C2	1.498 (4)
C7—C8	1.378 (4)	C1—N1	1.309 (4)
C7—H7	0.9300	C1—N2	1.364 (3)
C5—C4	1.389 (4)	N1—H11	0.79 (3)
C5—I1	2.100 (3)	N1—H12	0.82 (4)
S1—C1	1.680 (3)	C3—C4	1.379 (4)
O1—C9	1.232 (3)	C3—C8	1.402 (4)
N4—C9	1.350 (3)	C3—C2	1.457 (3)
N4—C8	1.399 (3)	N2—H21	0.83 (4)
N4—H41	0.77 (3)	C4—H4	0.9300
			
C7—C6—C5	121.0 (2)	C1—N1—H11	118 (2)
C7—C6—H6	119.5	C1—N1—H12	119 (3)
C5—C6—H6	119.5	H11—N1—H12	122 (4)
C6—C7—C8	117.5 (3)	C4—C3—C8	120.7 (2)
C6—C7—H7	121.2	C4—C3—C2	133.3 (3)
C8—C7—H7	121.2	C8—C3—C2	105.9 (2)
C6—C5—C4	121.3 (2)	N3—N2—C1	119.9 (2)
C6—C5—I1	117.67 (19)	N3—N2—H21	121 (2)
C4—C5—I1	121.0 (2)	C1—N2—H21	119 (2)
C9—N4—C8	111.2 (2)	C7—C8—N4	128.4 (3)
C9—N4—H41	122 (2)	C7—C8—C3	121.6 (2)
C8—N4—H41	127 (2)	N4—C8—C3	110.0 (2)
C2—N3—N2	116.5 (2)	N3—C2—C3	126.1 (2)
O1—C9—N4	127.1 (3)	N3—C2—C9	127.4 (2)
O1—C9—C2	126.5 (2)	C3—C2—C9	106.5 (2)
N4—C9—C2	106.4 (2)	C3—C4—C5	117.8 (2)
N1—C1—N2	117.0 (2)	C3—C4—H4	121.1
N1—C1—S1	125.4 (2)	C5—C4—H4	121.1
N2—C1—S1	117.5 (2)		
			
C5—C6—C7—C8	−0.1 (4)	C2—C3—C8—N4	−0.6 (3)
C7—C6—C5—C4	−0.3 (4)	N2—N3—C2—C3	179.7 (2)
C7—C6—C5—I1	−179.88 (19)	N2—N3—C2—C9	−2.1 (4)
C8—N4—C9—O1	−179.3 (2)	C4—C3—C2—N3	−1.1 (5)
C8—N4—C9—C2	−0.5 (3)	C8—C3—C2—N3	178.9 (2)
C2—N3—N2—C1	−178.7 (2)	C4—C3—C2—C9	−179.7 (3)
N1—C1—N2—N3	−4.7 (3)	C8—C3—C2—C9	0.3 (3)
S1—C1—N2—N3	176.33 (18)	O1—C9—C2—N3	0.4 (4)
C6—C7—C8—N4	−178.9 (2)	N4—C9—C2—N3	−178.4 (2)
C6—C7—C8—C3	0.3 (4)	O1—C9—C2—C3	179.0 (2)
C9—N4—C8—C7	−180.0 (2)	N4—C9—C2—C3	0.1 (3)
C9—N4—C8—C3	0.7 (3)	C8—C3—C4—C5	−0.4 (4)
C4—C3—C8—C7	0.0 (4)	C2—C3—C4—C5	179.5 (3)
C2—C3—C8—C7	−180.0 (2)	C6—C5—C4—C3	0.5 (4)
C4—C3—C8—N4	179.4 (2)	I1—C5—C4—C3	−179.85 (18)

### Hydrogen-bond geometry (Å, º)

**Table:**

*D*—H···*A*	*D*—H	H···*A*	*D*···*A*	*D*—H···*A*
N1—H12···N3	0.85 (4)	2.28 (4)	2.633 (3)	105 (3)
N2—H21···O1	0.83 (4)	2.07 (4)	2.725 (3)	135 (3)
N4—H41···O1^i^	0.77 (3)	2.04 (4)	2.809 (3)	178 (3)
N1—H11···S1^ii^	0.79 (3)	2.66 (4)	3.448 (3)	170 (3)

Symmetry codes: (i) −*x*+1/2, *y*−1/2, −*z*+3/2; (ii) −*x*, −*y*+4, −*z*+1.
